# Corpus callosum volumes in the 5 years following the first-episode of schizophrenia: Effects of antipsychotics, chronicity and maturation

**DOI:** 10.1016/j.nicl.2018.03.015

**Published:** 2018-03-15

**Authors:** Mariana T.M. de Moura, Marcus V. Zanetti, Fabio L.S. Duran, Maristela S. Schaufelberger, Paulo R. Menezes, Marcia Scazufca, Geraldo F. Busatto, Mauricio H. Serpa

**Affiliations:** a Laboratorio de Neuroimagem em Psiquiatria (LIM-21), Departamento e Instituto de Psiquiatria, Hospital das Clinicas HCFMUSP, Faculdade de Medicina, Universidade de Sao Paulo, Sao Paulo, SP, BR; b Nucleo de Apoio a Pesquisa em Neurociencia Aplicada (NAPNA), Universidade de Sao Paulo, Sao Paulo, SP, BR; c Laboratorio de Neurociencias (LIM-27), Departamento e Instituto de Psiquiatria, Hospital das Clinicas HCFMUSP, Faculdade de Medicina, Universidade de Sao Paulo, Sao Paulo, SP, BR; d Departamento de Medicina Preventiva, Faculdade de Medicina FMUSP, Universidade de Sao Paulo, Sao Paulo, SP, BR; e Laboratorio de Psicofarmacologia, Psicopatologia Experimental e Terapeutica Psiquiatrica (LIM-23), Departamento e Instituto de Psiquiatria, Hospital das Clinicas HCFMUSP, Faculdade de Medicina, Universidade de Sao Paulo, Sao Paulo, SP, BR

**Keywords:** AP, antipsychotics, CC, corpus callosum, FEP, first episode of psychosis, FESZ, First-episode of schizophrenia-related psychoses, GM, gray matter, MEM, mixed-effects model, ROI, region-of-interest, VBM, voxel-based morphometry, VolBM, volume-based morphometry, WM, white matter, Schizophrenia, Psychosis, Corpus callosum, White matter, Magnetic resonance imaging

## Abstract

**Background:**

White matter (WM) structural changes, particularly affecting the corpus callosum (CC), seem to be critically implicated in psychosis. Whether such abnormalities are progressive or static is still a matter of debate in schizophrenia research. Aberrant maturation processes might also influence the longitudinal trajectory of age-related CC changes in schizophrenia patients. We investigated whether patients with first-episode schizophrenia-related psychoses (FESZ) would present longitudinal CC and whole WM volume changes over the 5 years after disease onset.

**Method:**

Thirty-two FESZ patients and 34 controls recruited using a population-based design completed a 5-year assessment protocol, including structural MRI scanning at baseline and follow-up. The linear effects of disease duration, clinical outcome and antipsychotic (AP) use over time on WM and CC volumes were studied using both voxelwise and volume-based morphometry analyses. We also examined maturation/aging abnormalities through cross-sectional analyses of age-related trajectories of total WM and CC volume changes.

**Results:**

No interaction between diagnosis and time was observed, and clinical outcome did not influence CC volumes in patients. On the other hand, FESZ patients continuously exposed to AP medication showed volume increase over time in posterior CC. Curve-estimation analyses revealed a different aging pattern in FESZ patients versus controls: while patients displayed a linear decline of total WM and anterior CC volumes with age, a non-linear trajectory of total WM and relative preservation of CC volumes were observed in controls.

**Conclusions:**

Continuous AP exposure can influence CC morphology during the first years after schizophrenia onset. Schizophrenia is associated with an abnormal pattern of total WM and anterior CC aging during non-elderly adulthood, and this adds complexity to the discussion on the static or progressive nature of structural abnormalities in psychosis.

## Highlights

• Continuous exposure to antipsychotics is associated with increased corpus callosum.
• White matter presents a dysmaturational pattern in psychotic patients.
• Chronicity and clinical outcome do not influence white matter volumes.

## 1 Introduction

The neurobiology of schizophrenia is not yet fully elucidated. In the last decades, neuroimaging studies have reported several structural and functional brain abnormalities associated with the disorder (Innocenti et al., 2003; Arnone et al., 2008; Bora et al., 2011), some of which seem to be directly related to clinical course and symptom dimensions (Ellison-Wright and Bullmore, 2009; Bora et al., 2011; Asami et al., 2012; Jääskeläinen et al., 2014; Rosa et al., 2015). Among the findings of such investigations, convergent evidence of structural MRI studies has corroborated the existence of cerebral white matter (WM) abnormalities in schizophrenia (Steen et al., 2006; Walterfang et al., 2006; Ellison-Wright et al., 2008; Di et al., 2009; Ellison-Wright and Bullmore, 2009; Bora et al., 2011).

The WM contains myelinated axonal fibers that interconnect gray matter (GM) structures all over the brain, possessing a crucial role in the integration of neural information. In addition to structural MRI investigations, post-mortem studies found reductions in size and density of oligodendrocytes and abnormal myelin structure and compactness in the WM of schizophrenia patients, which may interact with synaptic abnormalities to produce disrupted brain connectivity (Davis et al., 2003; Walterfang et al., 2006). Such findings are in line with the notion that abnormal integration of brain networks is central to the neurobiology of schizophrenia, as postulated in the disconnection hypothesis (Davis et al., 2003; Walterfang et al., 2006; Schmitt et al., 2011; Kochunov and Hong, 2014).

A major subject of debate in schizophrenia research is whether the structural brain changes observed in MRI investigations are static or progressive over time. Progression of volumetric GM abnormalities has been consistently documented after the onset of schizophrenia, particularly in patients with a non-remitting course of illness (Bora et al., 2011; Vita et al., 2012; Haijma et al., 2013; Rosa et al., 2015). There is also evidence suggesting that WM abnormalities might be progressive in schizophrenia. One meta-analysis of longitudinal studies (1–10 years of follow up) using region-of-interest (ROI) analysis found significant reduction of frontal, temporal and parietal WM over time in schizophrenia, although no significant effect for total WM was observed (Olabi et al., 2011).

The corpus callosum (CC), which is the largest bundle of axonal fibers in the brain and the main commissure connecting the cerebral hemispheres, seems to be one of the most affected WM structures in psychosis (Keshavan et al., 2002; Hulshoff Pol et al., 2004; Arnone et al., 2008; Shepherd et al., 2012; Watson et al., 2012). Reductions of CC area or volume, mainly affecting its anterior portions, have been consistently described in schizophrenia, including first-episode patients (Keshavan et al., 2002; Hulshoff Pol et al., 2004; Arnone et al., 2008; Walterfang et al., 2009; Chaim et al., 2010). Also, smaller genu of the CC has been associated with conversion to psychosis in ultra-high risk (UHR) subjects (Walterfang et al., 2008a). However, to date only a few studies specifically examined the longitudinal course of CC abnormalities in adults with schizophrenia. Mitelman et al. (2009) found that patients with chronic schizophrenia had a faster decline in absolute CC size than healthy controls over 4 years of follow-up, and this effect was stronger in poor-outcome patients. Only one longitudinal investigation studied patients with first-episode of schizophrenia-related psychoses (FESZ) (n = 19) and found no differences relative to healthy controls (n = 19) in the rates of change of CC volumes over a 1-year follow-up period (Del Re et al., 2016). So far, no study has evaluated progression of CC volumes over a fairly long period of time after illness onset. Moreover, the potential impact of antipsychotic (AP) use and illness course/prognosis on CC volumes has not been investigated using a longitudinal design in the initial years of the disease.

As the brain is under constant change due to maturation and aging, another relevant question in neuroimaging research is whether findings of structural brain abnormalities in schizophrenia might actually be related to deviations in these processes. MRI investigations evaluating the age-related brain changes suggest that schizophrenia is associated with both a dysmaturational pattern and accelerated brain aging (Van Haren et al., 2008; Bose et al., 2009; Douaud et al., 2009; Koutsouleris et al., 2014; Kochunov et al., 2016). Studies with schizophrenia patients examining the effects of age on the WM are still scarce and inconclusive (Van Haren et al., 2008; Bose et al., 2009; Andreasen et al., 2011). Regarding the effects of age on the CC structure, one cross-sectional investigation found that, whereas healthy adults exhibited an age-related increase in total CC area, this pattern was absent in treatment-naïve FESZ patients (Keshavan et al., 2002). Nevertheless, it is important to notice that the authors assessed linear correlations only and more recent evidence suggests that the CC matures/ages in a nonlinear fashion (Lebel et al., 2012), which might have limited the sensitivity of their analyses.

In the present study, a population-based cohort of young adults with FESZ and epidemiological controls were studied with structural MRI scanning at baseline and after 5 years of naturalistic follow-up. The FESZ patients enrolled here are part of a larger sample of first-episode psychosis (FEP) that exhibited volumetric reductions affecting the right frontal WM and the genu and splenium of the CC at study entrance (Chaim et al., 2010; Colombo et al., 2012). We employed both voxel-based morphometry (VBM) and volume-based morphometry (VolBM) analyses aiming to examine: (1) The occurrence of volume changes affecting the CC over 5 years of follow-up in FESZ patients; (2) The potential influence of clinical course (i.e., sustained remission versus non-remission) and AP use in CC volumes over time; (3) Abnormalities in the trajectories of maturation/aging of the CC in FESZ patients. This is particularly relevant considering that nonlinear changes related to maturation/aging processes may obscure the investigation of both static and progressive brain differences between patients and controls. The same analyses were carried out for the total WM compartment in order to allow interpretation of findings in the CC in the light of comprehensive changes in brain WM over time.

We hypothesized that FESZ patients would present a global reduction of CC volumes over time, which might be more pronounced in the portions that presented reductions at baseline (anterior and posterior), as well as an accelerated age-related volume loss of the CC relative to controls. We also hypothesized that the longitudinal changes observed would be more pronounced in patients with non-remitting course.

## 2 Method

### 2.1 Participants

The FESZ patients examined herein were selected from a large sample of FEP individuals who took part in a population-based case-control study investigating the incidence of psychotic disorders in a circumscribed region of Sao Paulo city, as previously described (Menezes et al., 2007; Colombo et al., 2012). In the original epidemiological investigation, cases were identified by active surveillance of all people that made contact for the first time with the mental healthcare services attending that region (approximately 900,000 inhabitants) due to psychotic symptoms. In order to obtain a population-based control sample at the time of the baseline assessment, next-door neighbors were actively contacted and enrolled as volunteers (Menezes et al., 2007).

In Chaim et al. (2010) and Colombo et al. (2012), our group examined baseline differences in, respectively, CC and WM volumes between FEP and controls drawn from this cohort. In the present investigation, we opted to include only those patients who fulfilled criteria for a first-episode of schizophrenia, schizophreniform disorder or schizoaffective disorder (FESZ group) according to the Diagnostic and Statistical Manual for Mental Disorders, 4th edition (DSM-IV; American Psychiatric Association, 1994) and who completed the 5-year follow-up evaluation. A subsample of controls who completed the 5-year follow-up assessment, matched for age (within a range of 5 years) and gender to the FESZ group, was also drawn from the original control group.

Other inclusion criteria for both FESZ patients and controls at baseline were: (a) age between 18 and 50 years; (b) residing for 6 months or more in defined geographic areas of Sao Paulo. Exclusion criteria for both cases and controls at any time of the study were: (a) history of head injury with loss of consciousness; (b) presence of neurological disorders or any organic disorders that could affect the central nervous system; (c) moderate or severe mental retardation; and (d) contraindications for MRI scanning. Exclusion criteria specific for controls were: (a) diagnosis of any DSM Axis I disorders (except substance misuse or mild anxiety disorders); and (b) personal history of psychotic symptoms (lifetime), assessed with the Psychosis Screening Questionnaire (Bebbington and Nayani, 1995).

Following baseline evaluation, patients were referenced to treatment at the health services located in the geographical regions where they lived in. Both FESZ patients and controls included in the present study were followed-up naturalistically over a 5-year period, with re-interviews carried out for diagnostic confirmation and assessment of prognosis (remitting versus non-remitting course in the patients). From the original sample studied at baseline (Chaim et al., 2010; Colombo et al., 2012), 32 FESZ (22 with schizophrenia, 04 with schizophreniform disorder and 06 with schizoaffective disorder) and 34 controls completed the 5-year follow-up protocol (including a new MRI scanning) and were included in the present analyses. Information on data attrition can be found in Rosa et al. (2015) and in the Supplementary Table 1.

Local ethics committees approved this investigation and all participants provided informed written consent before the study assessments both at baseline and at the 5-year follow-up.

### 2.2 Clinical assessment

All participants (patients and controls) were evaluated both at baseline (T0) and at 5-year follow-up (T5) with the Structured Clinical Interview (SCID) for DSM-IV (First et al., 1995) for the assessment of psychiatric diagnosis. For FESZ patients, the presence and severity of psychotic symptoms was measured with the Positive and Negative Syndrome Scale (PANSS) (Kay et al., 1987). In addition, a general medical history and information about the use of psychotropic medications through medical records and interviews with patients and/or family was also obtained.

At 5-year follow-up, patients were categorized into remitting or non-remitting courses according to the DSM-IV course specifiers, assessed with the SCID: remitting course meaning a single psychotic episode in full remission, without new psychotic episode during the follow-up, and a non-remitting course meaning the presence of either continuous or episodic symptoms, or residual/negative symptoms. To investigate the effects of AP treatment on longitudinal WM changes, FESZ patients were divided into two groups based on AP use during the follow-up period: patients who had been on continuous and regular treatment, and those who had quit treatment with AP after the baseline evaluation.

### 2.3 MRI acquisition

Imaging data were acquired both at baseline and at follow-up using two identical 1.5 T MRI scanners (GE Signa, General Electric, Milwaukee, WI, USA). In order to test the reliability between devices, analyses of brain measures of six controls who were examined in both scanners in the same day were conducted, demonstrating a very good consistency between them (see Chaim et al., 2010 and Colombo et al., 2012 for further details); for instance, an intraclass correlation coefficient of 0.98 was observed for CC measures.

The same acquisition protocol was used in both scanners: a T1-spoiled gradient recall (SPGR) sequence providing 124 contiguous slices, slice thickness = 1.5 mm, matrix size = 256 × 192, echo time = 5.2 ms, repetition time = 21.7 ms, flip angle = 20. At baseline, all MRI scannings were collected with the same field of view (FOV = 22 cm), resulting in a voxel with the following dimensions: 0.86 × 0.86 × 1.5 mm. At the follow-up MRI scanning session, the same FOV and voxel dimension parameters were set initially, but the technical team that conducted the MRI acquisitions were allowed to adjust the FOV (from 22 to 28 cm) before image acquisition in cases where fittings were judged to be needed due to inter-subject differences in head size. Thus the final voxel sizes at the follow-up scanning session were: the same as initially set (0.86 × 0.86 × 1.5 mm) in 8 patients and 9 controls; 0.94 × 0.94 × 1.5 mm in 5 patients and 1 control; 1.02 × 1.02 × 1.5 mm in 16 patients and 22 controls, 1.05 × 1.05 × 1.5 mm in 2 patients, and 1.09 × 1.09 × 1.5 mm in 1 patients and 2 controls. No difference between groups regarding the distribution of voxel sizes of follow-up MRI acquisitions was observed (Likelihood Ratio = 6.973, df = 4, p = 0.137). All images were resampled to an isotropic voxel (1.5 × 1.5 × 1.5 mm) as part of the processing protocol adopted here (see below).

An experienced neuroradiologist visually inspected all images with the purpose of identifying artifacts during image acquisition and the presence of silent gross brain lesions.

### 2.4 MRI processing

VBM was performed using Statistical Parametric Mapping (SPM) 8.0 (http://www.fil.ion.ucl.ac.uk/spm/software/spm8), executed in Matlab platform (Mathworks, Sherborn, MA).

First, follow-up images were registered to baseline images and reoriented; the mm coordinate of the anterior commissure matched the origin xyz (0, 0, 0), and the orientation approximated Montreal Neurological Institute (MNI) space. Then, all images were segmented and classified into GM, WM and cerebrospinal fluid using the unified segmentation implemented in SPM8, which provides both the native space versions and Diffeomorphic Anatomical Registration Through Exponentiated Lie algebra (DARTEL) imported versions of the tissues (Ashburner and Friston, 2005). A customized template was created from the subjects using the DARTEL protocol (Ashburner, 2007; Matsuda et al., 2012). The deformation field was applied to the segmented images in sequence. Finally, the images created in the previous step were standardized to MNI space, re-sliced to 1.5 × 1.5 × 1.5 mm voxels and smoothed using an 8-mm full width at half maximum (FWHM) Gaussian kernel. The total WM volumes were obtained from the modulated images. The choice to use a 8 mm Gaussian filtering size in the current study was based on the fact that such degree of smoothing provides an optimal degree of increment in signal-to-noise ratio and conformation of MRI data to a normal distribution (thus allowing the use of parametric tests in subsequent statistical comparisons), as well as compensating for some of the data loss incurred by spatial normalization (Salmond et al., 2002; Mechelli et al., 2005).

**Fig. 1:**
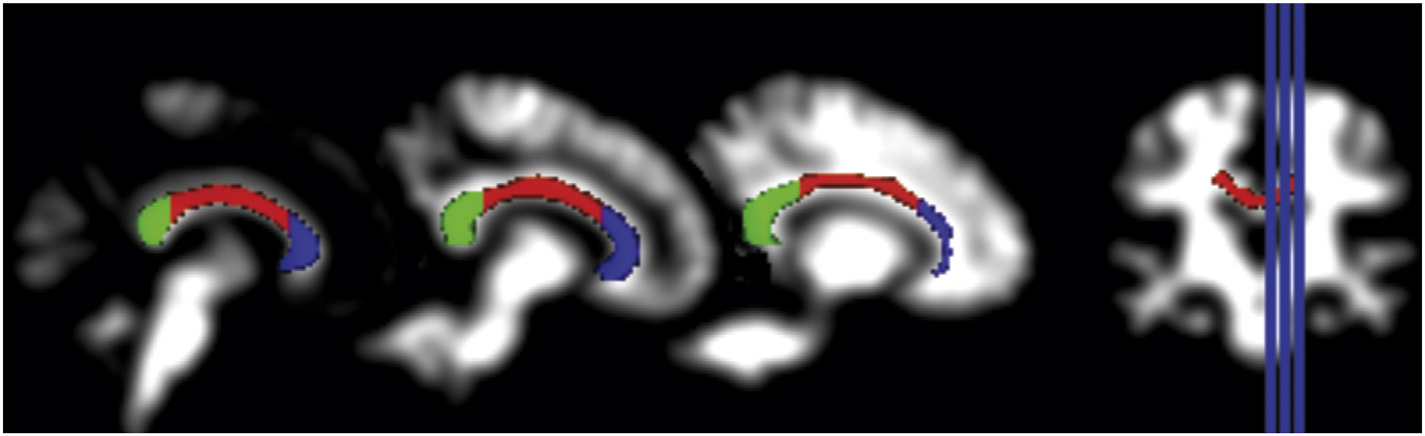
ROI masks derived from a DTI-based WM atlas (Mori et al., 2008) circumscribing CC. ROIs are underlaid by a T1 smoothed image of whole-brain WM compartment. CC main portions are differentiated by colors: anterior (dark blue), mid (red) and posterior (green). Note: ROI, region-of-interest; DTI, diffusion tensor imaging; WM, white matter; CC, corpus callosum.

Small Volume Correction (SVC) analyses were performed by masking the WM volumetric map with CC ROIs derived from a DTI (diffusion tensor imaging)-based WM atlas (Mori et al., 2008). By constraining the total number of voxels included in the analyses, the SVC approach improves the statistical power of hypothesis-driven analyses within specific CC regions. The predefined ROIs were projected onto each individual image to circumscribe total CC limits as well as to divide it on anterior, mid and posterior portions; such portions were arbitrarily defined to contain CC genu, body and splenium, respectively (Mori et al., 2008) (Fig. 1).

To further explore abnormal patterns of age-related volume change affecting the CC in FESZ, we also conducted VolBM analyses of total WM and CC volumes. Differently from the SVC method described above, here the volumes of total WM and of total, anterior, mid and posterior CC were extracted for each and every subject to be analyzed using statistical tests not available in the SPM8 package. The measure of total WM volume was obtained with the “get_totals” script (http://www.cs.ucl.ac.uk/staff/g.ridgway/vbm/get_totals.m) implemented for SPM8 on the native space of WM segmentation. For the CC measures, we applied the spatially normalized DTI-based ROI masks (described above) on each subject registered image and then extracted the volumes using a script from the SPM mailing list (https://www.jiscmail.ac.uk/cgi-bin/webadmin?A2=spm;3d3a3add.0809).

### 2.5 Statistical analyses

#### 2.5.1 Clinical and demographic data

Between-groups comparisons of demographic and clinical data were carried out using the Statistical Package for Social Sciences (SPSS, 20.0 version). We employed the Chi-square or Fisher's exact tests for categorical variables and the Student's *t*-test or an analysis of variance (ANOVA) for continuous variables. Statistical significance was set at p < 0.05, two-tailed.

#### 2.5.2 Voxelwise longitudinal analyses

A repeated-measures analysis of covariance (ANCOVA) contrast, with group and time as factors, was chosen to assess between-group differences in longitudinal WM changes. The following voxel-by-voxel comparisons of CC volumes (total and three main portions) were performed using the SPM8 statistical package:

– Total group of FESZ patients (n = 32) versus controls (n = 34);
– Remitted patients at T5 (n = 17) versus non-remitted patients at T5 (n = 15) versus controls (n = 34);
– Patients on continuous AP use at T5 (n = 18) versus patients without AP (n = 14) versus controls (n = 34).

Only voxels with values above an absolute threshold of p < 0.05 entered the analyses. A measure of the total amount of WM was entered as a covariate in all analyses, given by the sum of voxels within the corresponding WM compartment of each subject. Resulting statistics at each voxel were transformed to Z scores, and between-group differences were displayed as statistical parametric maps (SPMs) of the group x time interactions into standard anatomical space, thresholded at the one-tailed p < 0.001 level of statistical significance (corresponding to a Z > 3.09 threshold). As described above, the SVC tool of SPM8 was employed with the purpose of restricting the comparisons to voxels located within the CC and its three main portions (resulting in a search volume of 2674 voxel for the genu, 4076 voxels for the body and 3758 voxels for the splenium).

Clusters with a minimum of 10 contiguous voxels showing significant findings within each of those volumes of interest were reported only if they survived family-wise error (FWE) correction for multiple comparisons (p < 0.05) over that region (Friston et al., 1996). Significant ANCOVA findings were followed-up with two-group post-hoc *t*-tests. The same comparisons were repeated including age and gender as nuisance variables.

Subsequently, the SPM maps were inspected again on an exploratory basis aiming to identify possible regions of WM volume change over time in FESZ patients versus controls. Such findings were reported as statistically significant only if surviving FWE correction for multiple comparisons (p < 0.05) over the whole brain.

In all analyses, MNI coordinates from the voxel of maximal statistical significance were converted to the Talairach and Tournoux System (Lacadie et al., 2008).

#### 2.5.3 Mixed-effects model analyses of longitudinal data

Longitudinal changes in brain structure might be influenced by intra-individual factors; such random effects are not weighted by the statistical models commonly used in conventional voxelwise methods (Bernal-Rusiel et al., 2013; Mills and Tamnes, 2014). Mixed effects models (MEM) were devised to deal with this issue, thus being more sensitive to detect between-group differences that might be overshadowed by individual variability (Bernal-Rusiel et al., 2013; Mills and Tamnes, 2014). For this reason, MEM analyses were performed on the volumes of total WM and of total, anterior, mid and posterior CC extracted from the preprocessed images, as described above.

Linear MEM was run in the SPSS package, with total WM volume and each ROI of the CC as a dependent variable. In order to select the best fitting model, different models were tested using a three-step approach: first only fixed factors were included; then the random intercept was added; and lastly random slopes were included. For all analyses and steps, the fixed factors were: age, gender, group, time point, age X group, time point X group, and total WM volume (only when examining CC volumes). At step three, the interactions of age X group, and time point X group were also set as random slopes. Only for the FESZ group, MEM were performed including clinical status and AP use at T5, as well as its interaction with time point as fixed factors; at step three, both interactions were set as random slopes. In all analyses, values of p were corrected for multiple comparisons with the false discovery rate (FDR) approach.

#### 2.5.4 Cross-sectional analyses of maturation/aging patterns

For the evaluation of age-related trajectories of total WM and CC volumes change in FESZ patients and controls, the ROIs of total WM and of total, anterior, mid and posterior CC previously extracted from follow-up images were analyzed with SPSS 20.0. Regression analyses (“Curve Estimation” option) were used to determine the most appropriate trajectory model to describe the relationship between each ROI measurement (dependent variable) versus age (predictor) in each group. CC volumes were normalized by total WM volume. First, second and third order polynomial expansions were assessed and the best fitting models were reported.

## 3 Results

### 3.1 Sociodemographic and clinical characteristics

FESZ patients (n = 32) and controls (n = 34) did not differ regarding age, gender and interval between MRI scans. However, patients attained fewer year of education and had a higher rate of comorbid substance abuse/dependence compared to controls (Table 1), as commonly reported by other studies (Regier et al., 1990; Kavanagh et al., 2002; Mueser and McGurk, 2004; Barnett et al., 2007).

**Table 1:** Sociodemographic and clinical characteristics of FESZ patients and controls.

Characteristics^a^	FESZ (n = 32)	Controls (n = 34)	Statistical tests^b^		
Age at study entrance, years	28.2 (8.5)	30.8 (8.3)	t = −1.224	df = 64	p = 0.218
Gender, males	24 (75%)	19 (56%)	χ ^2^ = 2.654	df = 1	p = 0.126
Education, years	8.5 (3.9)	10.6 (4.5)	t = −2.017	df = 64	p = 0.048⁎
Interscan interval, months	60.2 (8.9)	59.3 (11.4)	t = 0.330	df = 64	p = 0.743
Substance abuse or dependence at follow-up, n	10 (31%)	1 (3%)	χ ^2^ = 9.512	df = 1	p = 0.002⁎
PANSS, positive score - T0	12.1 (6.1)				
PANSS, positive score - T5	10.3 (5.2)				
PANSS, negative score - T0	14.1 (6.59)				
PANSS, negative score - T5	11.9 (5.17)				
PANSS, total score - T0	51.0 (14.0)				
PANSS, total score - T5	44.1 (14.5)				
Clinical course, n – remmited	17 (53%)				
On regular treatment during follow-up, n	21 (65.6%)				
Confirmed clinical diagnosis (T5)					
Schizophrenia	22 (68.8%)				
Schizophreniform disorder	4 (12.5%)				
Schizoaffective disorder	6 (18.7%)				

Note: FESZ, first-episode of schizophrenia-related psychosis. Df, degrees of freedom. PANSS, Positive and Negative Syndrome Scale. T0, baseline. T5, follow-up of 5 years.

a Continuous variables are expressed as mean and standard deviation; categorical variables are expresses as frequency and %.

b Continuous variables were analyzed with the *t*-test for two samples. Categorical variables were analyzed with the Pearson chi-square test (and with Fisher exact test when necessary).

⁎ Significant statistical difference (p < 0.05).

FESZ patients who remained remitted (n = 17) and those with a non-remitting course over the follow-up period (n = 15) had similar clinical characteristics, including interval between MRI scans, frequency of substance use disorder and exposure to AP in days (Supplementary Material - Table 2). Patients in use of AP (n = 18) and those not using AP (n = 14) at follow-up had similar characteristics as well (Supplementary Material - Table 2).

### 3.2 VBM analyses

#### 3.2.1 CC and its subregions

No significant longitudinal differences were observed between the whole group of FESZ subjects versus control group in regard to total CC volumes (total volume and of its anterior, mid and posterior portions). Also, there were no significant differences in the comparison between remitted FESZ patients at T5 versus non-remitted FESZ patients at T5 versus controls on the change of CC volumes over time.

**Fig. 2:**
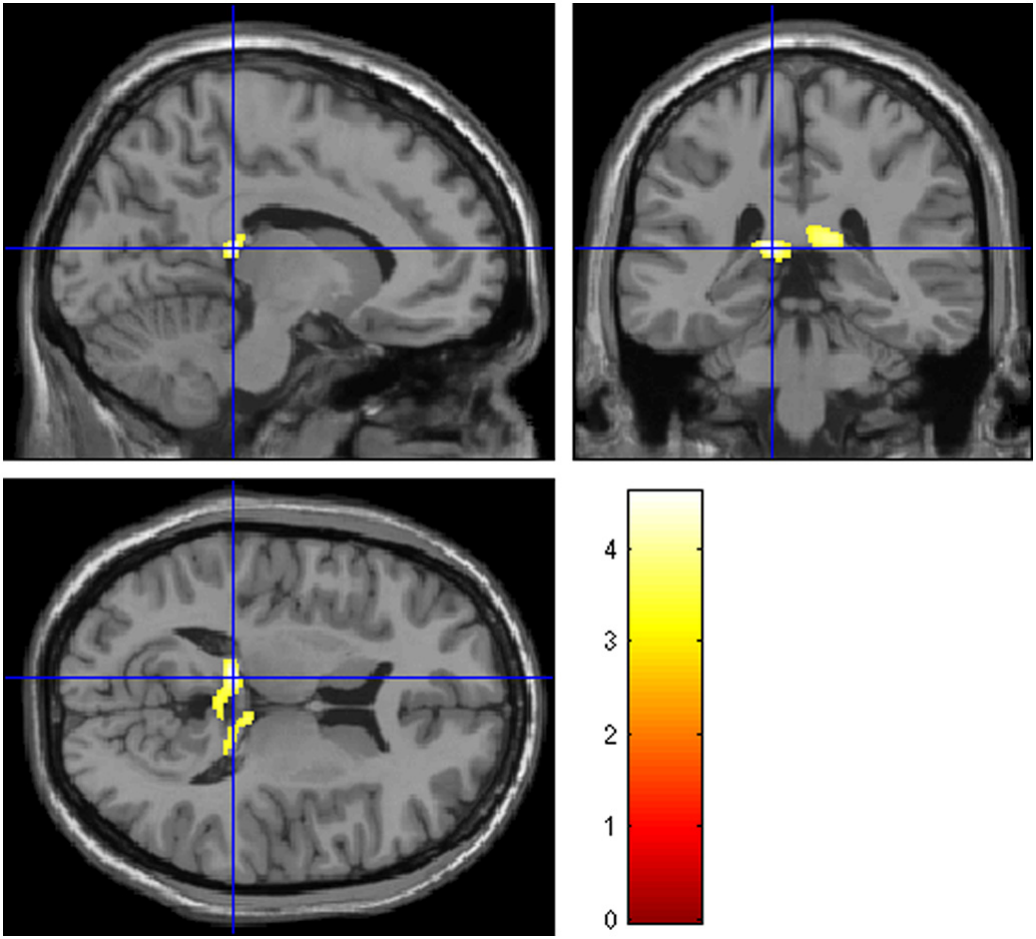
Cluster of significant WM increase in FESZ patients continuously using AP medication over 5 years of follow-up in comparison to controls, located in the posterior region of CC, involving both brain hemispheres (MNI coordinates: X = −11, Y = −34, Z = 12, cluster size = 390 voxels, peak Z score = 4.17). ANCOVA, p < 0.05 FWE corrected. Color bar represents Z scores. Note: WM, white matter. FESZ, first-episode of schizophrenia-related psychosis. AP, antipsychotics. CC, corpus callosum. FWE, family-wise error.

Nevertheless, FESZ patients on continuous AP use showed greater volumetric increase over time in the posterior CC relative to controls, in a cluster involving both brain hemispheres (pFWE = 0.014; peak Z score = 4.17; MNI coordinates: X = −11, Y = −34, Z = 12; cluster size = 390 voxels) (Fig. 2). Results did not change significantly when the analyses were repeated including age and gender as confounding variables/fixed factors. No longitudinal differences were observed for total, anterior and mid CC volumes.

To explore the influence of including total WM volume as a covariate in the final results, we repeated the voxelwise comparisons without controlling for total WM volume, which likely increases the sensitivity of the analyses. After repeating the voxelwise comparison without controlling for total WM volume, the following results emerged:

– The negative result regarding longitudinal differences between the whole group of FESZ subjects and controls in CC volumes remained unchanged;
– FESZ patients on continuous AP use also showed greater volumetric increase over time in the posterior CC relative to controls (pFWE = 0.003; peak Z score = 4.25; MNI coordinates: X = −11, Y = −34, Z = 12; cluster size = 556 voxels);
– One additional cluster of greater volumetric increase was observed in the anterior CC of FESZ patients on continuous AP use relative to controls (pFWE = 0.042; peak Z score = 3.44; MNI coordinates: X = 11, Y = 20, Z = 15; cluster size = 60 voxels);
– Finally, non-remitted FESZ patients showed greater volume increase over time in both posterior (pFWE = 0.021; peak Z score = 3.75; MNI coordinates: X = 5, Y = −40, Z = 13; cluster size = 453 voxels) and anterior CC (pFWE = 0.050; peak Z score = 3.38; MNI coordinates: X = 5, Y = 30, Z = 0; cluster size = 31 voxels) relative to controls.

These results did not significantly change when the analyses were repeated including age and gender as confounding variables/fixed factors.

#### 3.2.2 Whole-brain WM volumes

In the voxelwise comparison between the whole group of FESZ patients and controls, no significant differences in regional WM volumes over time were observed. Also, clinical status of FESZ patients (remitting versus non-remitting course versus controls) as well as AP use (continuous AP use versus no AP use versus controls) at follow-up did not significantly influence longitudinal changes in WM volumes, i.e. no significant differences emerged in these comparisons.

### 3.3 Mixed-effect models analyses of CC and total WM volumes

For total WM and total CC volumes, the best fitting model based on Akaike's Information Criteria was determined using a heterogeneous AR(1) (Heterogeneous First-Order Autoregressive) structure of variance-covariance matrix and including only fixed effects (i.e., random intercepts and random slopes have not contributed for the goodness of fit). For anterior and mid CC volumes, the best fitting model was determined using an AR(1) (First-Order Autoregressive) structure of variance-covariance matrix and including only fixed effects. Regarding the posterior CC volume, the best fitting model was determined using a heterogeneous AR(1) (Heterogeneous First-Order Autoregressive) structure of variance-covariance matrix, and adding random intercept and slopes to the model reduced its error (covariance structure of random effects: identity; p = 0.088).

No significant interaction between age and group or time point and group was observed for any of the dependent variables tested (see Table 2 for all MEM results). For total WM, gender (t = 4.762, pFDR<0.001, male as reference) and time point (t = −3.285, p-FDR = 0.005) were significant determinants of total WM volumes. For total, anterior, mid and posterior CC volumes, only total WM was a significant factor (respectively: t = 7.936, pFDR<0.001; t = 6.842, pFDR<0.001; t = 8.872, pFDR<0.001; and t = 6.725, pFDR<0.001).

**Table 2:** Total WM and CC volumes in FESZ patients and controls: linear mixed-effects analyses, estimates of fixed effects.

Whole sample	Intercept	Age	Gender	Time point	Total WM	Group	Age × group	Time point × group
Total WM	**502.57427**	−0.210486	**56.847017**	**−13.1634**	–	55,045,774	−1.901,908	7,842,737
CC	4,634,238	0.020830	0.037537	0.103703	**0.031399**	1,813,646	−0.076247	0.284240
CC anterior region	0.138775	−0.004761	−0.125906	0.049346	**0.010772**	0.256867	−0.011928	0.017027
CC mid region	1,397,698	0.000864	0.007650	0.156248	**0.013445**	0.677782	−0.026599	0.101712
CC posterior region	**2.166246**	0.014872	0.026835	−0.020573	**0.009584**	0.611840	−0.028452	0.126078


FESZ	Intercept	Age	Gender	Time point	Total WM	Clinical course	Time point × clinical course	Time point × continuous AP use
Total WM	**586.767076**	**−2.698553**	35,004,856	−5.179,548	–	2,872,321	4,778,318	3,603,932
CC	−0.398630	−0.033944	−0.247376	0.423576	**0.042659**	1,262,453	0.097237	0.021070
CC anterior region	−1.779,282	−0.007464	−0.167129	0.081865	**0.014270**	0.225019	0.009133	−0.021361
CC mid region	1,462,156	−0.023673	−0.130371	0.246324	**0.014405**	0.497733	−0.012995	0.049781
CC posterior region	0.281298	−0.006340	0.054761	0.098719	**0.013470**	0.534861	0.106607	0.004229

Note: WM, white matter. CC, corpus callosum. FESZ, first episode of schizophrenia-related psychosis. AP, antipsychotics.

: p < 0.001 for T-test of estimates; : p < 0.005 for t-test of estimates; : p < 0.05 for *t*-test of estimates.

All results were corrected for multiple comparisons (false discovery rate correction, FDR). Gender: male as reference; Group: FESZ as reference; Clinical Course: remission as reference.

In the analyses restricted to the FESZ group, the same pattern of model fitting was observed, with the exception of the posterior CC portion, for which the best model was determined by an AR(1) (First-Order Autoregressive) structure of variance-covariance matrix and including only fixed effects. No significant interaction between clinical course and time point or AP intake and time point was observed for total WM or CC volumes (see Table 2). For total WM, age was the only significant predictor (t = −3.188, pFDR <0.05), indicating that the older the patient, the smaller the volume of brain WM. For total, anterior, mid and posterior CC volumes, similarly to what was observed for the whole sample, total WM was the only significant determinant (respectively: t = 8.233, pFDR<0.001; t = 5.717, pFDR <0.001; t = 10.665, pFDR<0.001; and t = 6.737, pFDR<0.001). Also, for mid CC volume, a strong tendency (t = 2.551, pFDR = 0.056) was observed for time point, pointing to increasing volumes over time in the FESZ group.

### 3.4 Aging trajectories of WM and CC volumes

**Fig. 3:**
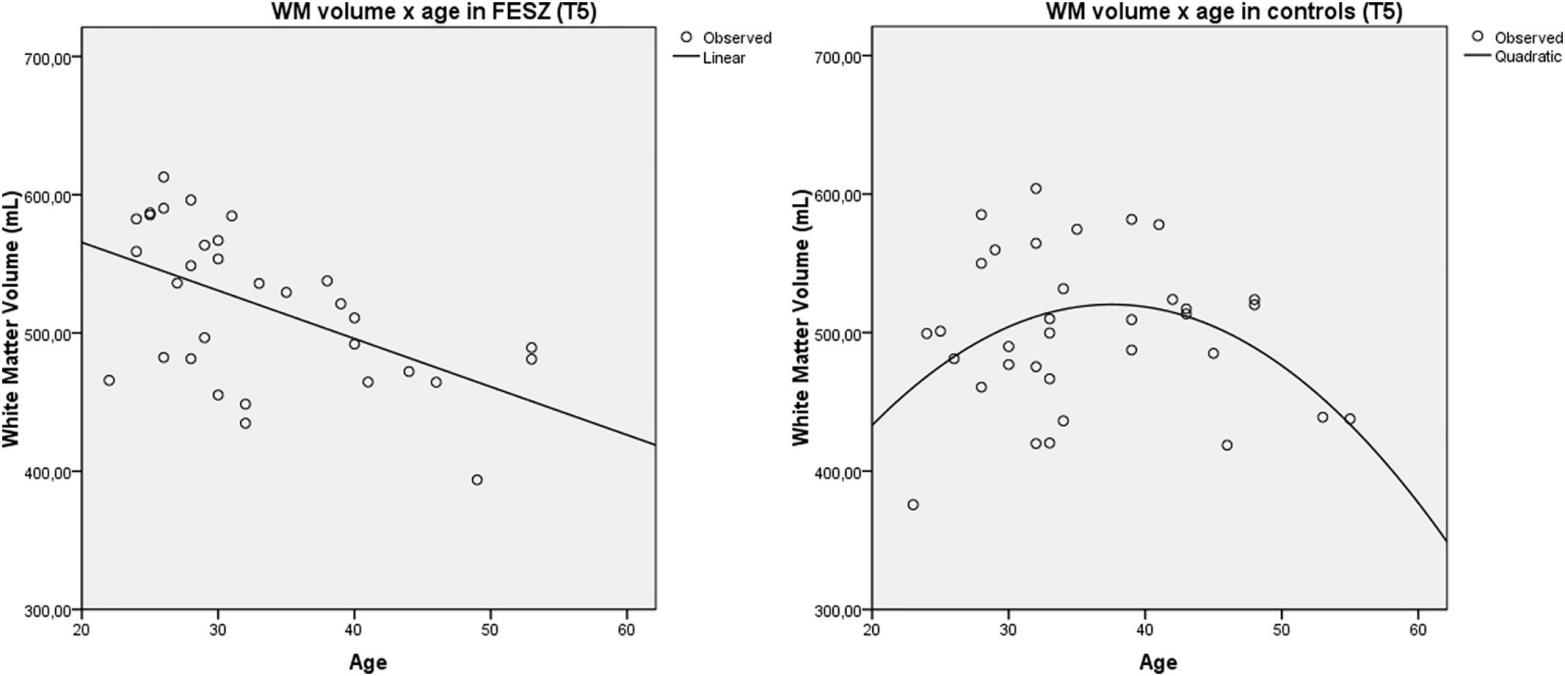
Plots of WM volumes versus age in FESZ (a) and controls (b) at T5, including best fitting regression curves. Note: WM, white matter. FESZ, first-episode of schizophrenia-related psychosis. T5, follow-up evaluation.

**Fig. 4:**
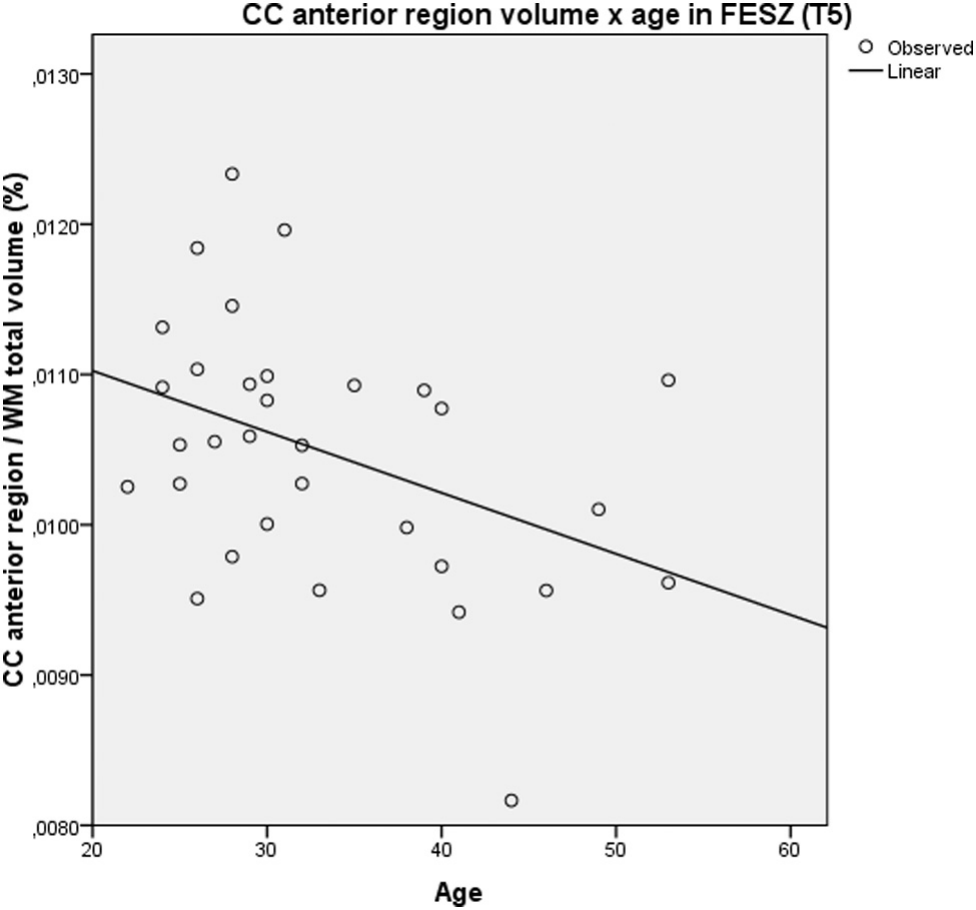
Plots of CC anterior region volumes versus age in FESZ sample at T5, including best fitting regression curve. CC region volumes were corrected for the total amount of WM. Note: WM, white matter. CC, corpus callosum. FESZ, first-episode of schizophrenia-related psychosis. T5, follow-up evaluation.

In FESZ patients, a pattern of linear WM volume decline with age was observed (R^2^ = 0.293, p = 0.001). Differently, controls showed a trend toward a non-linear pattern of WM aging, with total volume increase until the fourth decade of life and volumetric loss from then onwards (R^2^ = 0.146, p = 0.087) (Table 3 and Fig. 3). A significant linear volumetric loss with age was found in the anterior CC region of FESZ patients (R^2^ = 0.156, p = 0.020), whereas relative volume preservation during non-elderly adulthood was observed in mid and posterior CC portions of FESZ subjects and in all CC portions of controls (Table 3 and Fig. 4). These results were obtained by evaluating the volumes extracted from follow-up (T5) images; nonetheless, the same pattern of findings emerged when we analyzed volumes extracted from baseline (T0) images.

**Table 3:** Trajectory of WM and CC aging/maturation in FESZ patients and controls.

VOI	FESZ (n = 32)			Controls (n = 34)		
	Best fitting model	R^2^	p	Best fitting model	R^2^	p
WM	Linear	0.293	0.001	Quadratic	0.146	0.087
Total CC	–			–		
CC anterior region	Linear	0.156	0.020	–		
CC mid region	–			–		
CC posterior region	–			–		

Note: WM, white matter. CC, corpus callosum. FESZ, first episode of schizophrenia-related psychosis. VOI, volume of interest.

Best fitting polynomial regression models by brain region. First, second and third order polynomial expansion were assessed (only regions with significant or trend findings are shown).

In order to better interpret our results on CC maturation and to increase sensitivity, we conducted post-hoc analyses for the trajectories of CC without controlling for total WM. The best fitting curve for all CC volumes (total, anterior, mid, and posterior) of FESZ patients was represented by a linear reduction of volumes by age (all significant; see Supplementary Material – Table 4). For controls, no fitted curve was statistically significant.

## 4 Discussion

To our knowledge, this is the first structural MRI study to examine longitudinal morphometric changes in the CC of FESZ patients over the first 5 years after disease onset, as well as to assess age-related trajectories of CC volume change in schizophrenia patients. In the present investigation, while no significant changes in CC volumes were observed in FESZ patients relative to controls, continuous AP use was associated with greater increase of the posterior CC region over the 5 years of follow-up. Also, the regression analyses focusing on age-related effects on total WM and CC volumes found a different maturation/aging pattern in FESZ patients, who presented a linear decline of total WM and anterior CC with age, whereas controls showed an inverted U-shape pattern for total WM volume and relative preservation of CC volumes during non-elderly adulthood.

Differently from our initial hypothesis, no significant changes over time in CC volumes were observed in FESZ patients relative to controls in voxelwise analyses using the SVC approach. Previous studies investigating the effects of illness course on CC morphometry in schizophrenia yielded variable results. In a meta-analysis of 28 cross-sectional investigations, CC area reductions were found to be more pronounced in FEP patients, while patients with chronic schizophrenia exhibited relatively greater CC area (Arnone et al., 2008). The few longitudinal studies published so far mostly showed findings in a opposite direction, reporting reductions of CC measures over time (Keller et al., 2003; Mitelman et al., 2009), particularly in patients with poor prognosis (Mitelman et al., 2009). However, these studies have evaluated chronic schizophrenia patients and employed CC midsagittal area as the main measure. The only longitudinal study published to date to assess CC volumes in FESZ also failed to find significant changes over a 1-year follow-up period compared to controls (Del Re et al., 2016). It is important to notice that the repeated-measures ANCOVA contrast used for voxelwise comparisons here and the statistical analyses conducted by Del Re et al. (2016) are based on linear statistics, and would likely not detect nonlinear patterns of volumetric changes over time. Nevertheless, these findings suggest that no progression of CC volume deficits occur in the first years after schizophrenia onset.

We also failed to find significant longitudinal changes in regional WM volumes in the whole-brain voxelwise comparison between FESZ and control groups, which was corroborated by the MEM analyses.

Contrary to our finding, one large longitudinal investigation of FESZ reported reductions in total, frontal, temporal and parietal WM of patients over time, which were more pronounced in the first years of follow-up (Andreasen et al., 2011). One possible explanation for the negative result observed here is that the population-based approach used to recruit participants for the present investigation could have led to the enrollment of cases with milder severity of illness than in previous longitudinal studies (Keller et al., 2003; Mitelman et al., 2007). Interestingly to this regard, clinical status of FESZ patients (i.e., remitting versus non-remitting course) at T5 did not influence CC volumes over the 5 years of follow-up. Nonetheless, due to the modest size of our study groups, we cannot fully rule out the possibility of type II statistical errors.

On the other hand, as illness severity seems to be linked to the degree of brain changes over time (Mitelman et al., 2009; Andreasen et al., 2011), the fact that we studied milder cases of FESZ may have improved our sensitivity to detect effects of AP exposure in WM morphometry. Our VBM comparisons showed that FESZ patients on continuous AP use had greater increase in posterior CC relative to controls over time. The effects of AP agents on GM volumes have been well documented by several studies (Moncrieff and Leo, 2010; Vita et al., 2012; Fusar-Poli et al., 2013; Torres et al., 2013; Vita et al., 2015), but less is known regarding the impact of such medications in the WM. In a study with chronic and FEP patients, Molina et al. (2005) found that exposure to AP lead to decreases in parietal and occipital WM volumes. Ho et al. (2011) reported an association between AP intake and volume reductions in total, frontal, temporal and parietal WM in a large cohort of FEP. Bartzokis et al. (2011), in contrast, found increased frontal WM in chronic schizophrenia patients receiving long-acting risperidone. Regarding the CC, a cross-sectional study with FESZ and chronic schizophrenia patients did not find significant correlations between AP load and CC measures (Walterfang et al., 2008b). In the only longitudinal investigation of Del Re et al. (2016), no significant correlation was observed between rate of CC change over time and load of AP intake in FESZ. However, the very small size of study groups and the relatively short period of follow-up (1 year) of Del Re et al. (2016) work might have limited its sensitivity to detect CC volume changes related to AP use.

Regarding WM microstructure, DTI studies on the effects of AP are also inconclusive, reporting both increases and reductions in anisotropy following exposure to medication (Wang et al., 2013; Reis Marques et al., 2014; Szeszko et al., 2014; Ebdrup et al., 2016; Zeng et al., 2016; Serpa et al., 2017). Animal studies investigating the effects of exposure to AP on brain WM volumes are rare: in macaques, exposure to haloperidol and olanzapine caused non-significant decrements of parietal WM (Dorph-Petersen et al., 2005); in rodents, exposure to the very same AP did not influence CC volumes (Vernon et al., 2011).

One should consider that AP drugs actions on brain tissue might vary depending on medication class. Some studies have indicated a differential protective effect of atypical versus typical AP on brain volume changes in schizophrenia (Miyamoto et al., 2005; Ho et al., 2011; Goghari et al., 2011). Regarding the WM, Ho et al. (2011) demonstrated that higher doses of non-clozapine atypical AP were associated with enlarged parietal WM volumes over time. Another study observed that schizophrenia patients previously treated with typical AP and that were switched to olanzapine had a 25%-increase in the anterior internal capsule volume relative to controls in a 1-year follow-up (Goghari et al., 2011). In this regard, due to the naturalistic follow-up design of our study, we could not control our analyses in terms of neuroleptic type and lifetime load; thus, we cannot rule out that the exposure to a specific profile of AP type might have modulated our findings.

As molecular pathology in schizophrenia is still under investigation, effects of AP on brain structure are difficult to interpret. A possible explanation would be the restoration of damaged myelin, as *postmortem* investigations indicate that schizophrenia is associated with myelin/oligodendrocyte abnormalities (Takahashi et al., 2011) and animal and co-culture studies have demonstrated a potential role for AP in oligodendrocyte regeneration and myelin repair (Xiao et al., 2008; Zhang et al., 2008, Zhang et al., 2012; Bi et al., 2012; Chandran et al., 2012; Seki et al., 2013; Wang et al., 2015). Also, it is proposed that WM volume changes observed over aging in healthy individuals are caused by myelination/demyelination (Liu et al., 2014). Therefore, one could postulate that the reversal of pathological processes could be indirectly observed from longitudinal morphometric studies, which would be demonstrated by the stability or even the regression of reductions in WM volumes in patients continuously exposed to AP medication, as previously proposed (Arnone et al., 2008).

Our results for the voxelwise comparisons without controlling for total WM reinforce our initial findings that the chronic exposure to medication induces the enlargement of volumes over time. We also found new clusters showing increasing volumes over time in patients with poor-prognosis. Such clusters seem to overlap with the clusters found for continuous AP use, although they are smaller and present lesser statistical significance. It is conceivable that patients with worse prognosis should receive more AP medication over time; in fact, we observed a non-significant association between continuous use of neuroleptic medications and non-remission status (see Supplementary Material – Table 2). In other words, there is some collinearity between the exposure to AP drugs and poor clinical outcome. Therefore, we consider that such new findings that emerged when we conducted the analyses without controlling for total WM are possibly false-positives, indirectly reflecting the effect of the exposure to AP medication rather than a true relationship between poorer prognosis and larger CC volumes.

Another issue that should be considered in the interpretation of our results is the possibility of brain maturation abnormalities over the lifespan: when comparing FESZ patients to controls, differences in the trajectories of WM aging may obscure the examination of longitudinal changes directly associated to the disorder. Also, brain maturation might follow non-linear patterns, which are not detected by the statistical models generally used in MRI investigations.

Indeed, our analyses of aging effects on total WM and CC volumes pointed to an abnormal maturation/aging pattern in FESZ. In our study, controls showed a non-linear trend of WM maturation and aging, with an ascending curve until the fourth decade of life and volumetric loss subsequently (R^2^ = 0.146, p = 0.087). Despite not reaching statistical significance, such finding is in accordance with a number of previous studies on WM maturation in healthy volunteers that demonstrated quadratic curves with a peak in mid to late adulthood (Bartzokis et al., 2001; Jernigan et al., 2001; Allen et al., 2005; Westlye et al., 2010; Lebel et al., 2012; Liu et al., 2016). In contrast, FESZ subjects showed a linear decline of total WM volume with age (R^2^ = 0.293, p = 0.001). Employing a different metric, another 5-year follow-up study also observed an abnormal pattern of WM aging in schizophrenia, showing a faster decline in WM enlargement naturally observed in early adulthood (Van Haren et al., 2008). Bose et al. (2009), investigating solely linear differences in WM maturation in schizophrenia, found an accelerated WM loss with age in patients, in accordance with Andreasen et al. (2011), who demonstrated that FESZ patients present faster WM reductions in total, frontal, temporal, and parietal regions with aging. Therefore, our results reinforce such previous findings indicating that abnormal aging of brain WM in schizophrenia patients might be present since the first outbreak of the disease.

Evidence suggests that the maturation and normal aging of the CC and its sub-regions in healthy subjects are also non-linear during young adult life, showing the same inverted U-shape pattern as for total WM (Hasan et al., 2008; Prendergast et al., 2015; Liu et al., 2016). Schizophrenia seems to also disturb such normal process. A previous study examining the effect of age on callosal thickness and area demonstrated that chronic schizophrenia patients in the early adulthood showed a reduction of callosal thickness with age, in opposition to the expected expansion observed in healthy controls (Walterfang et al., 2008b). Another cross-sectional investigation showed that the expected expansion of CC area with age that occurred in controls was not observed in FEP patients (Keshavan et al., 2002). Nevertheless, so far no study has specifically evaluated non-linear volumetric trajectories of the CC in individuals with schizophrenia. In our study, in the analyses controlling for total WM, FESZ patients showed a linear reduction in anterior CC volume with age (R^2^ = 0.156, p = 0.020); when absolute volumes were examined, all CC measures showed linear reduction with age. Thus, CC seems to follow the pattern observed for global WM in patients, with even more accelerated volume reductions in its anterior part. Conversely, controls displayed overall volumetric stability of CC during non-elderly adulthood; this was also confirmed by the examination of absolute volumes. It is conceivable that pathological process affecting brain WM in schizophrenia might produce the same effect on all brain regions, indistinctly, causing widespread accelerated shrinkage of WM volumes. Regarding controls, no clear pattern was detected. Perhaps the restricted number of subjects might have precluded the observation of the normal non-linear maturational pattern; also, the short age range evaluated in our study might have limited our analyses, as CC aging seems to peak in early adulthood and remain stable till late life (Liu et al., 2016). In summary, the patterns observed in our study are in line with previous morphometric investigations of CC and aging in schizophrenia (Keshavan et al., 2002; Walterfang et al., 2008b).

The present investigation evaluated an enriched sample of FESZ, based on an epidemiological design with a naturalistic follow-up. Such approach aimed to control for unmeasured environmental effects (as patients and controls lived in the same region of Sao Paulo city) and to favor the recruitment of a group of FESZ that was more representative of community, in opposition to commonly used convenience samples of patients assisted in tertiary services. Notwithstanding, some limitations of this study may have influenced our results and should also be weighted in the interpretation of our findings. Firstly, as considered above, the final sample of FESZ patients and controls who completed the 5-years follow-up protocol can be considered modest, which increases the risk of both type I and II statistical errors. Therefore, our results may not represent trends of the overall schizophrenia spectrum, which is highly heterogeneous, but they might be limited to specific groups of patients. Secondly, a substantial proportion of our patients (30%) present comorbid substance misuse, which can affect WM and CC volumes (Chaim et al., 2010; Colombo et al., 2012). Lastly, we have included patients whose psychosis onset was relatively late in life (fifth decade of life). This might have added to the heterogeneity in our analyses, as the neurobiological mechanisms rendering vulnerability for developing psychosis might change with older age.

## 5 Conclusion

Our longitudinal investigation demonstrated that chronic exposure to AP medication is related to greater increase in posterior CC region in FESZ patients over time. Our analyses also revealed dysmaturational pattern in FESZ patients, who presented a linear decline of total WM and anterior CC volumes with age, while controls had a non-linear pattern of total WM maturation/aging and volumetric stability in the CC. Differences on brain maturation may interfere on the longitudinal effects of the disease, therefore obscuring conclusions on the static versus progressive issue. Replication of the present findings with a larger sample of patients is warranted.

The following are the supplementary data related to this article.

Supplementary Material - Table 1 Sociodemographic and clinical characteristics of FESZ patients which completed follow-up evaluation (T5) and of FESZ dropouts. Supplementary Material - Table 1

Supplementary Material - Table 2 Sociodemographic and clinical characteristics of FESZ patients according to remission status. Supplementary Material - Table 2

Supplementary Material - Table 3 Sociodemographic and clinical characteristics of FESZ patients according to AP use over follow-up. Supplementary Material - Table 3

Supplementary Material - Table 4 Trajectory of CC aging/maturation in FESZ patients and controls without controlling for total WM. Supplementary Material - Table 4

## Acknowledgments and financial support

This study was supported by the American Psychiatry Association award “APA/AstraZeneca Young Minds in Psychiatry International Award” to Dr. Maristela S. Schaufelberger (2007) and FAPESP scholarships no. 2014/00481-1 and 2013/03905-4 to M.T.M.M. and M.V.Z., respectively. Baseline MRI data was collected with the support of the Wellcome Trust (UK). M.H.S. receives a research scholarship from the Centro de Aperfeiçoamento de Pessoal de Nível Superior (CAPES, Brazil).
